# Supplementary material for: Photoinduced modulation of the oxidation state of dibenzothiophene S-oxide molecules on an insulating substrate
Source: Nat Commun. 2025 May 24;16:4841. doi: 10.1038/s41467-025-60075-y (PMC12103517; doi:10.1038/s41467-025-60075-y)
Supplement: Supplementary file 1 — Supplementary Information [file 41467_2025_60075_MOESM1_ESM.pdf]

Supplementary information for the article:
“Photoinduced Modulation of the Oxidation State of Dibenzothiophene *S*-Oxide Molecules on an Insulating Substrate”
by M. Hankache, V. Magné, *et al.*

Table of contents

I. On-surface Investigations 2

1. Particle probe models 2

2. Morphology of the substrate 4

3. High-resolution imaging of the supramolecular phase after UV-irradiation 5

4. Statistical analysis of the deoxygenation yield over time 7

5. Statistical analysis of the influence of the oxidation state on neighboring molecules . 13

6. DFT calculations of the (*o*-Br)$_2$-DBT and the pristine molecule (*o*-Br)$_2$-DBTO 15

7. Signature of the deoxygenation by spectroscopic CPD measurements 16

II. In-solution Investigations 19

1. Molecule synthesis 19

2. Photoreactivity in solution 20

3. NMR spectra 23

III. Crystallographic data 27

References 30

I. On-surface Investigations

1. Particle probe models

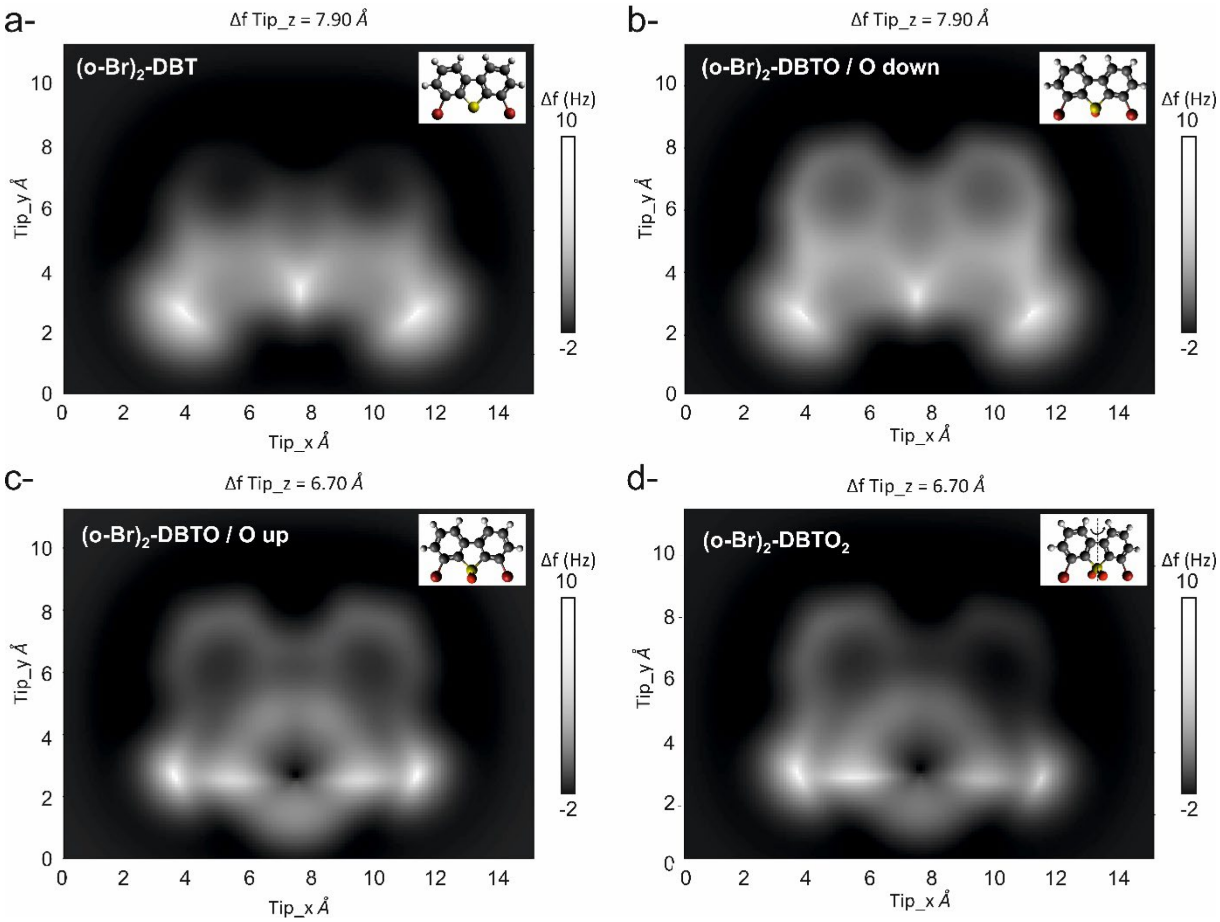

Fig. S1 | Particle probe model of the adsorption configuration of pristine (*o*-Br)$_2$-DBTO (images b,c) and deoxygenated (*o*-Br)$_2$-DBT (image a) molecules, as well as oxidized (*o*-Br)$_2$-DBTO$_2$ molecule (image d).

In solution, the photoreactivity of **(*o*-Br)$_2$-DBTO** molecules results in deoxygenated molecules (**(*o*-Br)$_2$-DBT**, 47% yield) and the oxidized byproduct **Br$_2$-OTO** (16%), as seen in Fig. 1. The latter **Br$_2$-OTO** displays a characteristic six-membered ring as central core, that would be clearly identifiable on high-resolution nc-AFM images. Such scaffold has never been observed experimentally on the surface, which allows to conclude that **Br$_2$-OTO** is not formed upon photoirradiation of **(*o*-Br)$_2$-DBTO** on surface. To understand how the **(*o*-Br)$_2$-DBTO** molecule is initially adsorbed on the surface, a comparison between experimental images and AFM simulated images of pristine molecules with either oxygen down (image **b**) or up (image **c**) was drawn. Alternatively, determining which final product is present on the surface, with respect to the in-solution photoreaction, is possible by comparing experiments with simulated images (**a**) and (**d**). The simulated constant height AFM images were obtained using the Probe Particle Model (PPM).[1,2] A CO-functionalized tip was selected as the type of probe, consistently with the experiments. The four simulated images allow comparisons between three different chemical structures: deoxygenated molecule **(*o*-Br)$_2$-DBT** (**a**), oxidized molecule **(*o*-Br)$_2$-DBTO$_2$**

(**d**) and sulfoxide precursors **(*o*-Br)$_2$-DBTO** with the oxygen atom pointing down (i.e. toward the surface, **b**) or pointing up (**c**). The structure and the orientation of each molecule are depicted in the white inset at the top right corner of each image. A small rotation of the structural model for an improved view of the **(*o*-Br)$_2$-DBTO$_2$** molecule is indicated by the black arrow in the inset of image (**d**). The C, H, O, S, and Br atoms are represented by gray, white, red, yellow, and burgundy spheres, respectively. The molecular structures used for PPM simulations (xyz files) were designed and optimized using Avogadro.[3] The mean molecular structures are planar to the substrate, and, once imported into PPM, are automatically shifted so that the topmost atom of the compound is positioned with an offset δ= -1.0 Å below zero. Therefore, the displayed simulated images are at different distances. However, the distance between the tip and the carbon backbone of the molecule is always the same. To understand that better, we detail the four configurations (**a** to **d**). The carbon scaffold of those compounds being essentially planar, the carbon atoms of compounds **a** and **b** (deoxidized **(*o*-Br)$_2$-DBT** and oxidized **(*o*-Br)$_2$-DBTO** with the oxygen pointing towards the sample, respectively) are at nearly equivalent vertical positions which correspond to the topmost position within the molecule. Corresponding PPM simulations are performed at an arbitrary, but similar, height of z = 7.9 Å, *i.e.* 8.9 Å above the carbon scaffold of each compound. Conversely, the topmost atoms in compounds **c** and **d** (oxidized **(*o*-Br)$_2$-DBTO** with the oxygen pointing upwards and **(*o*-Br)$_2$-DBTO$_2$**, respectively) are oxygen atoms, which are 1.2 Å above the mean carbon scaffold. Thus, to have a consistent 8.9 Å distance to the carbon scaffold the PPM simulations are performed at a height z = 8.9 Å - 1.2 Å + δ= 6.7 Å.

PPM simulations show that models (**a**) and (**b**) have a quite similar contrast. Models (**c**) and (**d**) have a quite similar contrast too, but significantly different compared to models (**a**) and (**b**). A notable repulsive contrast around the oxygen due to the significant oxygen-oxygen repulsion present in both the molecule and the tip now occurs. Upon comparing the simulated and experimental models (see Fig. S3b), the model with the oxygen down matches well the experimental images before illumination, indicating that the **(*o*-Br)$_2$-DBTO** molecule is initially adsorbed with its oxygen facing the surface. Following illumination, model (**a**) was identified on the surface, whereas model (**d**) (*i.e*. **(*o*-Br)$_2$-DBTO$_2$**) and **Br$_2$-OTO** were never observed. This indicates that, in the present case, the photoreaction on the surface only results in deoxygenation, thus yielding **(*o*-Br)$_2$-DBT** as a product.

2. Morphology of the substrate

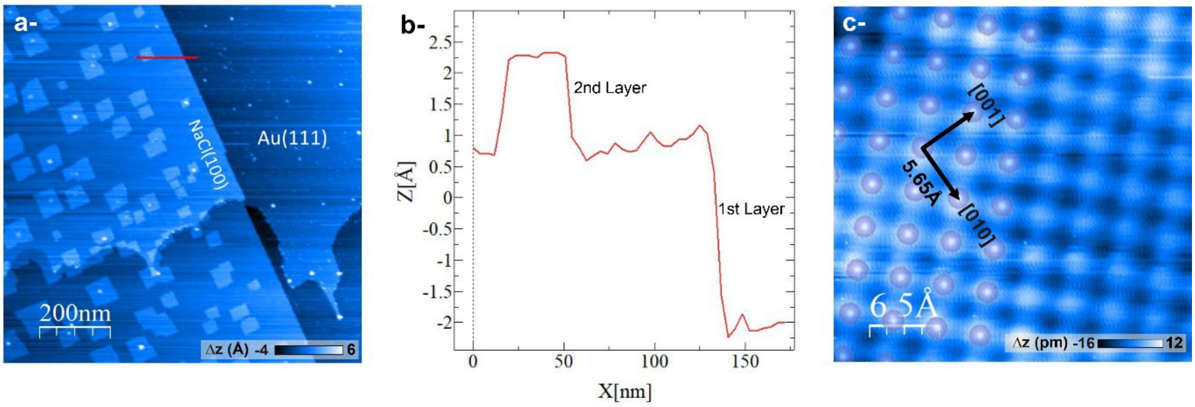

Fig. S2 | Surface investigation following the deposition of NaCl thin layers on Au(111). a, Large-scale STM image after the deposition of ~ 1 ML of NaCl onto Au(111) held at 450 K. The initial NaCl(100) layer exhibits a nearly carpet-like growth, followed by the formation of the second and third layers, as small rectangular islands. Tunneling parameters are I_t= 12 pA and V_b= -1.2 V. b, The red curve represents the height profile extracted from (a). This profile indicates that the first NaCl layer has an apparent height of (2.8 ± 0.2) Å, whereas the apparent height of the second rectangular layer corresponds to roughly (1.8 ± 0.2) Å. This is well-known with this type of surface and results from the increase of the tunnel barrier.[4] c, Atomically resolved STM image of the NaCl first layer on Au(111). The ionic structure's cations are overlaid as purple spheres. Tunneling parameters are I_t= 10 pA and V_b= 2.1 V.

3. High-resolution imaging of the supramolecular phase after UV-irradiation

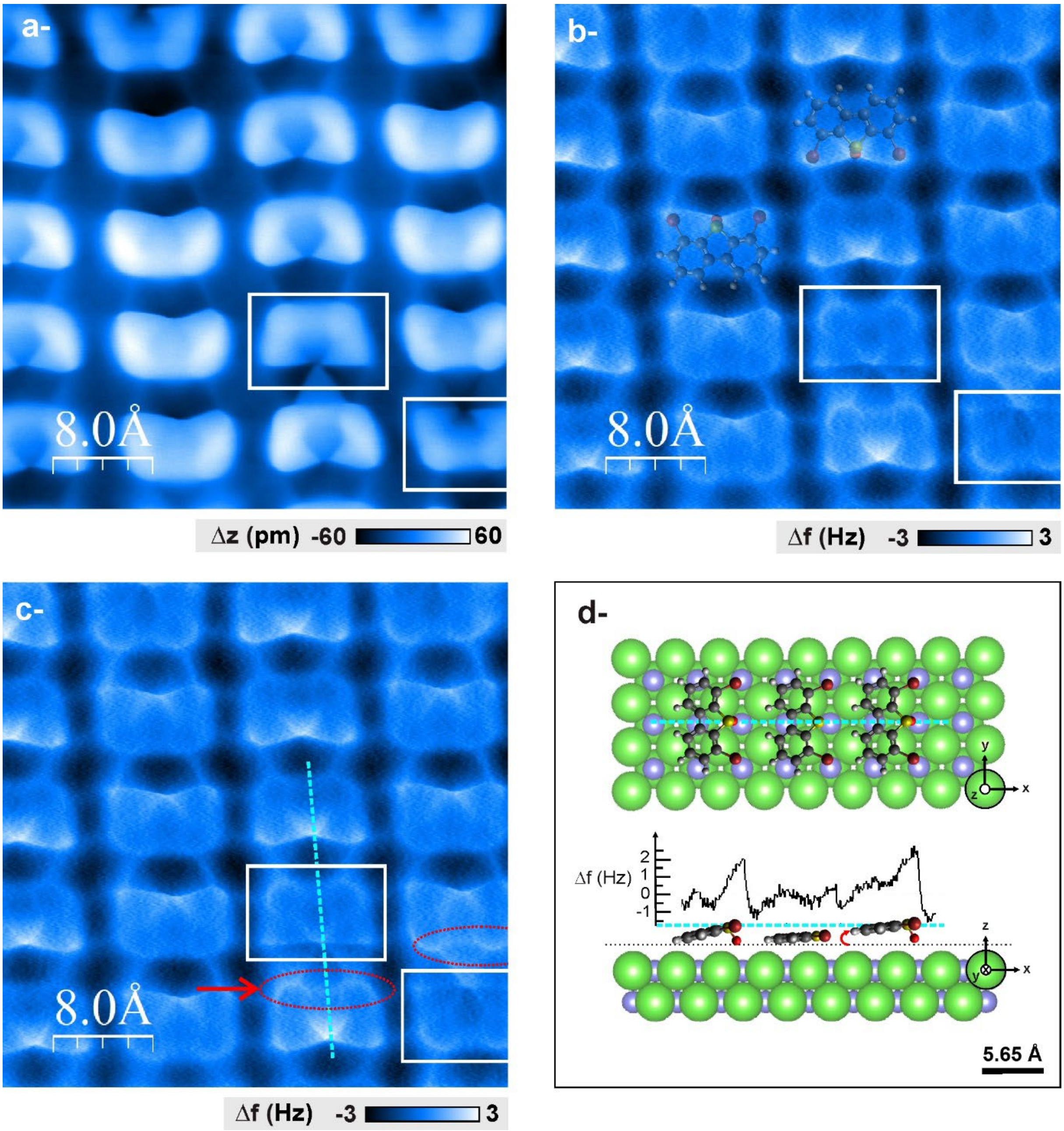

Fig. S3 | Dynamic STM observation. (<I_t>= 9.4 pA; V_b= 1.5 V and A= 150 pm) a, A typical dynamic STM image of the molecules on NaCl thin layers following UVA irradiation. The image shows 4 columns of molecules where at least two molecules are already deoxygenated and depicted by the white frames. b, Corresponding recorded map of frequency shift variation. Two structural models of (*o*-Br)$_2$-DBTO molecules, in two opposite orientations, are shown to be precisely matched with the contrast. A small change in the contrast around the sulfur atom is observed as the molecules flatten out on the surface due to deoxygenation, as shown in the white frames. A small contrast variation is also seen in

the following molecule when compared to a deoxygenated molecule. The red dotted ellipses in (c) and the red arrow in (d) indicate a slight elevation of the carbon skeleton of the molecule, which is actually the cause of this contrast. Fig. S3d is a model that was created for greater clarity. As seen in (c), the blue line represents the profiles of three consecutive molecules. According to the profile, on top of the sulfur atom of the deoxygenated molecule the Δf is -2 Hz lower than that on the sulfur atom of the pristine molecule, hence more attractive. This can once more draw attention to the differences between two distinct molecular states observed on the surface.

4. Statistical analysis of the deoxygenation yield over time

A statistical study was performed to evaluate the deoxygenation yield of adsorbed molecules as a function of illumination time. Following each illumination phase, we captured multiple images, each containing approximately 450 molecules, from different regions of the sample. The deoxygenation yield was then determined for each image using two independent methods, both giving consistent results. The first method, presented in section a, involved visually comparing the contrast of molecules to distinguish between deoxygenated and intact ones. The second method (section b), a more objective approach, served to validate the initial findings and eliminate any uncertainty.

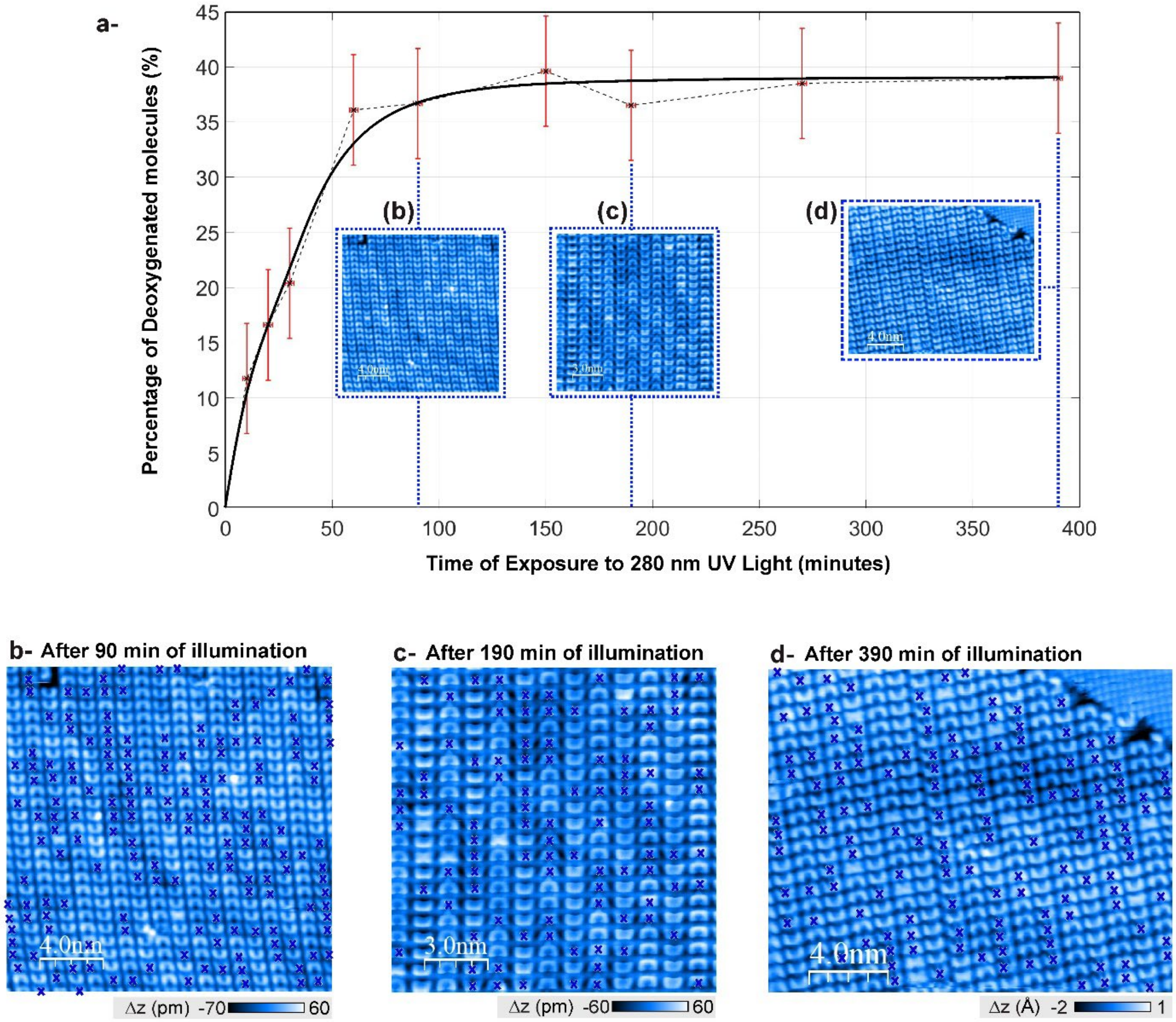

Fig. S4 | Statistical analysis of the deoxygenation yield as a function of illumination time. a, Percentage of deoxygenated molecules versus time of exposure to 280 nm UV light. b-d, Images of the supramolecular regime captured at different UV exposure durations: b, 90 minutes, c, 190 minutes, and d, 390 minutes. Deoxygenated molecules detected throughout the process are marked with a blue '*x*' symbol. Imaging parameters are b (I_t= 8 pA and V_b= 1 V), c ($\langle I_t \rangle$= 9.4 pA; V_b= 1.5 V and A= 150 pm), d (I_t= 8 pA and V_b= 1.5 V).

The study was conducted over 390 minutes. We estimate the uncertainty in the yield of deoxygenated molecules to be 10% (standard error). The data reveals a rapid increase in the deoxygenation yield, reaching 30% upon 50 minutes illumination, followed by a saturation of around 40%. To prevent overwhelming with excessive data, Fig. S4 highlights only three stages of the process, represented by images taken at different illumination times (**b**, 90 minutes, **c**, 190 minutes, and **d**, 390 minutes). On each image, deoxygenated **(*o*-Br)$_2$-DBT** molecules are marked with a blue ***x*** symbol.

Below, we will focus on the statistical analysis procedure, utilizing as an example the image captured at 190 minutes of illumination. The two distinct analysis techniques are presented for this image (visual interpretation on Fig. S5 and Software-automated interpretation on Fig. S6). The same procedure was applied to all other images at different time points.

a- <u>Visual Interpretation</u>

a-

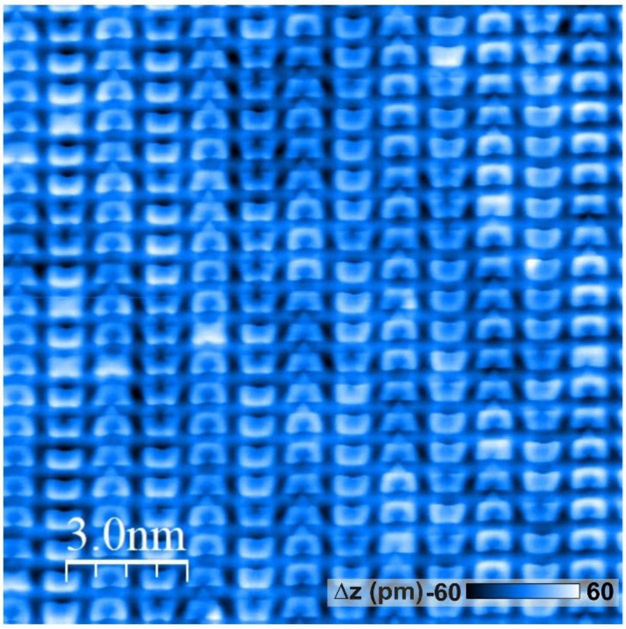

b-

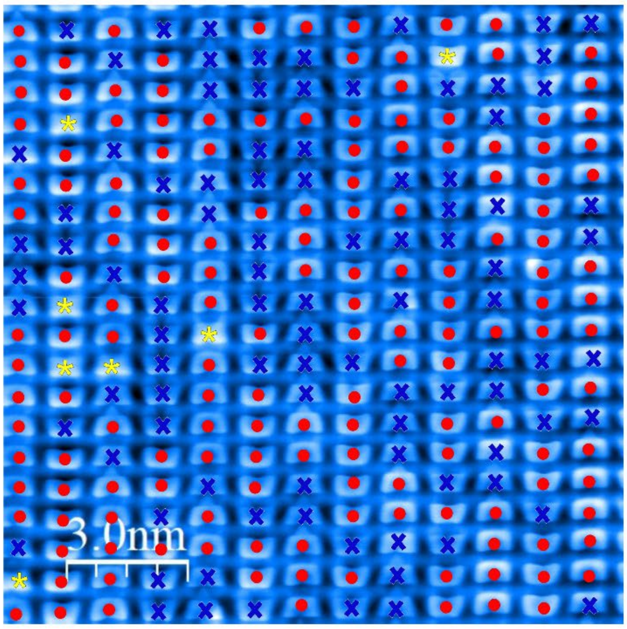

Fig. S5 | Statistical analysis following 190 minutes of UV irradiation. A total of 260 molecules exposed to UV irradiation (280 nm) underwent statistical analysis. a, High-resolution dynamic STM image with a CO-functionalized tip showing the molecules: <I_t>= 9.4 pA; V_b= 1.5 V and A= 150 pm. b, •, *x* and * symbols were used on the same image to denote the various molecular structures and/or configurations. The pristine (*croissant*-shaped) (*o*-Br)$_2$-DBTO molecules, which make up 60.7% of the total, are marked with the red • symbol. The deoxygenated (*butterfly*-shaped) (*o*-Br)$_2$-DBT molecules, of which there are 36.5%, are marked with the blue *x* symbol. Lastly, the yellow * represents a class of molecules that were present before illumination and have a different contrast, most probably due to a defect in either the NaCl thin film or the underlying substrate.

b- Software benchmarking method

This study provides an objective and efficient option for the statistical analysis of molecular modifications. It aims to create and validate an automated method to detect and classify molecules in supramolecular networks by examining SPM images. The code is scripted under MATLAB and consists of detecting individual molecules and calculating a similarity factor by cross-correlation with different predefined reference images corresponding to different states of the molecule. Large data sets may be examined using this approach, which makes it easier to quickly and objectively analyze molecular changes in supramolecular systems.

At first, we must provide an image of the matrix of molecules, for this example, we take the image used in the previous section (Fig. S6a). The network is built of molecules organized in alternating rows with different orientations. To account for potential biases resulting from the irregular shape of the STM tip and the natural arrangement of molecular rows, the reference dataset (Fig. S6b) contains two illustrative images of each molecular state (deoxygenated, pristine, and molecules with different contrast, from top to bottom, respectively), captured from both orientations of the rows (Fig. S6b). To increase molecular discernibility, the raw STM image is filtered (Fig. S6c) to remove electronic corrugation emanating from the underlying Au(111) surface. Then, the as-filtered image is transformed into a binary form (Fig. S6d), allowing it to efficiently isolate single molecules. The identified molecules then go through a cropping process, removing those located at the image boundaries to ensure a homogeneous size of each cropped area for subsequent analysis. Indeed, the frame size must match that of the reference molecules (Fig. S6b) and will be used to crop each molecule on the main unfiltered image (Fig. S6a) to calculate the cross-correlation. Each fitted molecule is compared with the reference set by adopting a minimum correlation threshold of 80%, thus a similarity score is determined. The classification is therefore performed by assigning to each molecule the state corresponding to the reference molecule with the highest similarity score. The final classification results, presented in Fig. S6f, illustrate the efficiency of the automated process. The algorithm identified 36.1% of deoxygenated **(*o*-Br)$_2$-DBT** molecules, showing a good match with the 36.5% obtained by visual interpretation, thereby validating the accuracy and reliability of the latter.

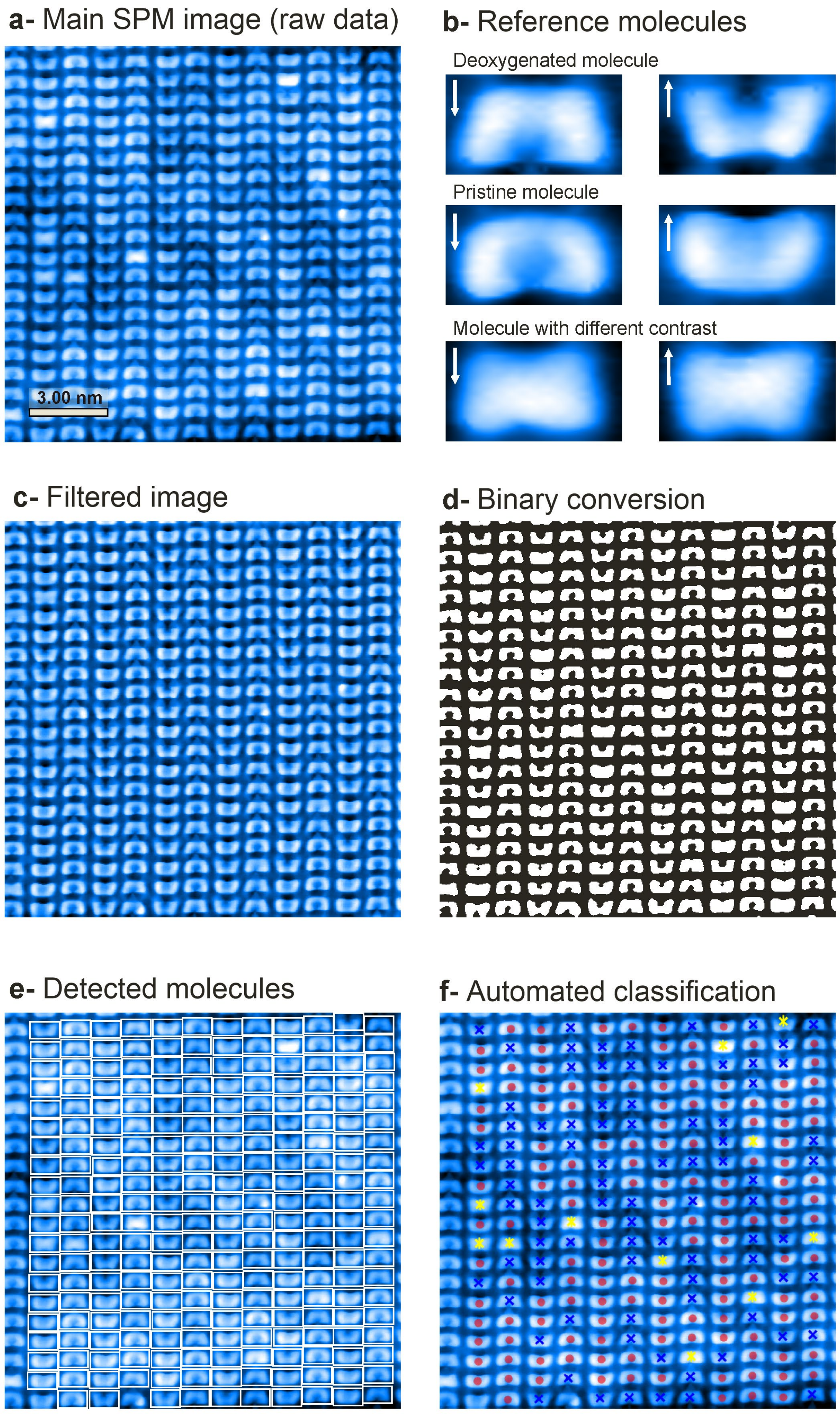
a- Main SPM image (raw data)
3.00 nm
b- Reference molecules
Deoxygenated molecule
Pristine molecule
Molecule with different contrast
c- Filtered image
d- Binary conversion
e- Detected molecules
f- Automated classification

Fig. S6 | Key steps for automated molecule detection and classification on an SPM image. a, Main STM image of the network formed by assembled molecules in alternating rows with different orientations. b, Reference images of molecules in different states (deoxygenated, pristine, and molecules with different contrast, from top to bottom, respectively) considering both row orientations (2 images on the same row correspond to the same state). c, Filtered image to remove Au(111) surface herringbone corrugation enhancing molecule detection. d, Binary conversion of image (c) to isolate individual molecules. e, Detected molecules with white rectangular bounding boxes of the same size as the reference images. Molecules whose bounding boxes go around the border of the main image are not selected for statistical study. f, Automated classification resulting from the comparison of each molecule detected with the reference images by cross-correlation. Molecules are automatically marked with color-coded labels similar to Fig. S5. Statistical automated results after 190 minutes UV irradiation: deoxygenated (*o*-Br)$_2$-DBT 36.1 %, intact (*o*-Br)$_2$-DBTO 58.8 %, and molecules with different contrast 5.0 %.

5. Statistical analysis of the influence of the oxidation state on neighboring molecules

The statistical distribution of deoxygenated **(*o*-Br)$_2$-DBT** molecules is studied using a MATLAB script. The experimental result in Fig. S5, with a deoxygenation yield of 36%, is converted to a 20 x 13 binary matrix where detected modified molecules are represented by one and pristine **(*o*-Br)$_2$-DBTO** molecules are represented by zero. To determine the influence of deoxygenated **(*o*-Br)$_2$-DBT** molecules on surrounding molecules, an analysis of the oxidation state of the surrounding molecules of every molecule was performed. Considering a deoxygenated **(*o*-Br)$_2$-DBT** molecule as the center element of a 3 x 3 matrix, the surrounding molecules are categorized based on their locations as directly front and rear, left and right, and diagonals of the center molecule. The percentage distribution of deoxygenated **(*o*-Br)$_2$-DBT** molecules (shown with blue bars in Fig. S7) and pristine **(*o*-Br)$_2$-DBTO** molecules (shown with red bars in Fig. S7) for each category of the surrounding molecules are recorded and presented in a grouped bar chart shown in Fig. S7a. The same analysis is done for surrounding molecules of every pristine **(*o*-Br)$_2$-DBTO** molecule and shown in Fig. S7b. Similarly, the statistical distribution was calculated for a simulated data set consisting of 50 randomly distributed matrices, as shown in Figs. S7c,d. Here, the neighboring deoxygenated molecules are homogeneously distributed around each molecule, according to the global deoxygenation yield of 36% and with a standard deviation of ~3% for such matrix size.

The experimental data of Figs. S7a,b show that the distribution of modified molecules is in first approximation close to the global yield of 36% whatever the neighboring location, thus indicating that the deoxygenation reaction occurs mostly randomly within the self-assembled layer. When looking into more details to the results, Fig. S7a shows that about 40% of molecules directly front and rear from deoxygenated molecules are themselves deoxygenated. This ratio is slightly higher than the global deoxygenation yield of 36%. In fact, we found systematically a distribution higher by about 5 points of deoxygenated molecules in the front and rear position of deoxygenated molecules compared to the global deoxygenation yield. Although this discrepancy is close to the standard deviation expected, this result can be put in perspective with the change in AFM contrast observed in Fig. S3. It is thus probable that the structural modifications induced in a molecule neighboring a deoxygenated molecule in rear position provides a slightly higher reaction probability upon UV irradiation.

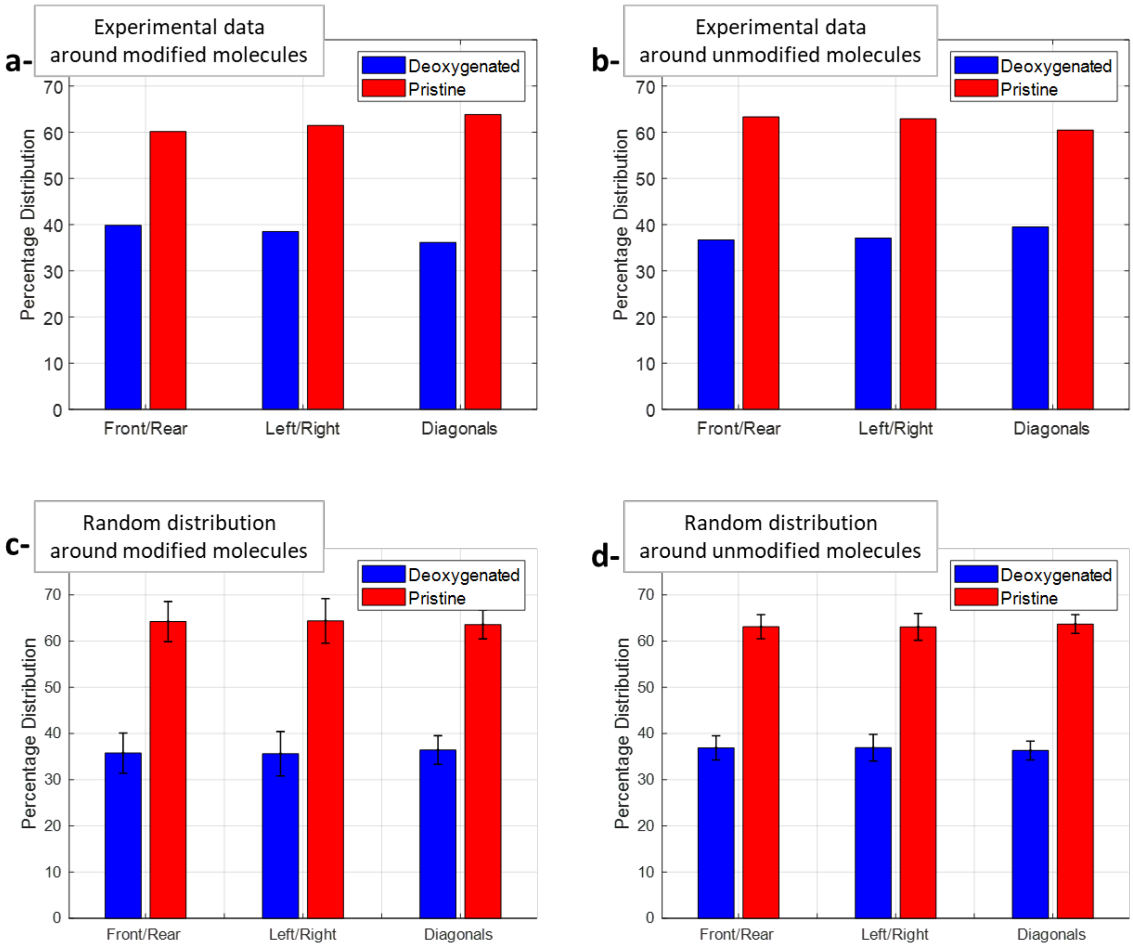

Fig. S7 | Statistical analyses of deoxygenation distribution as a function of surrounding molecules for a deoxygenation yield of 36%. Grouped bar chart of percentage distribution of deoxygenated molecules (blue bars) and pristine molecules (red bars) surrounding: deoxygenated (*o*-Br)$_2$-DBT (a) and pristine (*o*-Br)$_2$-DBTO (b) molecules from experimental data, deoxygenated (*o*-Br)$_2$-DBT (c) and pristine (*o*-Br)$_2$-DBTO (d) molecules from 50 randomly simulated matrices.

6. DFT calculations of the (*o*-Br)$_2$-DBT and the pristine molecule (*o*-Br)$_2$-DBTO

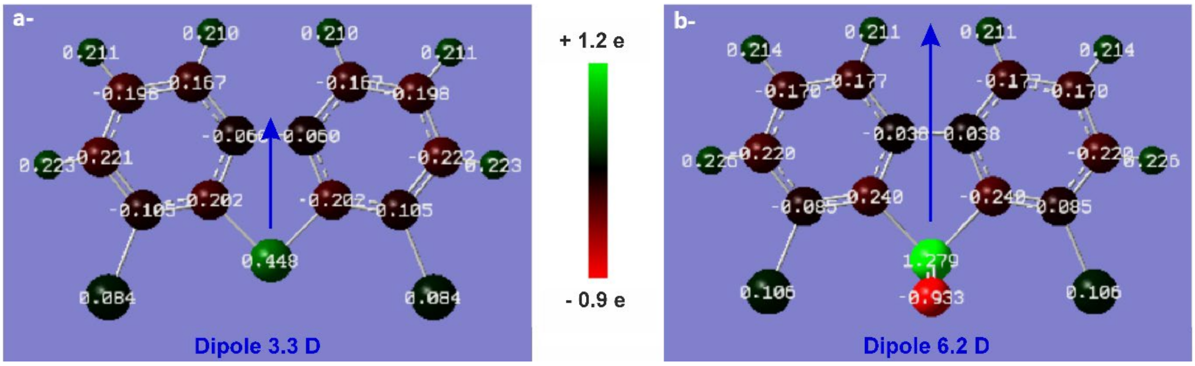

Fig. S8 | Density functional theory (DFT) calculations of charge distribution for (a) the deoxygenated molecule (*o*-Br)$_2$-DBT, and (b) the pristine molecule (*o*-Br)$_2$-DBTO. Natural Bond Orbital (NBO) analysis method was performed. The charge distribution scale ranges from red (negative) to green (positive). Calculations show that the sulfur atom of the deoxygenated (*o*-Br)$_2$-DBT molecule (a) bears a less positive charge (0.448) than the sulfur atom of the pristine form (*o*-Br)$_2$-DBTO (1.279). (b), in line with the change in sulfur oxidation state. The blue arrows indicate the dipole moment of each molecule: 3.3 D for (*o*-Br)$_2$-DBT molecule compared to 6.2 D for (*o*-Br)$_2$-DBTO molecule.

7. Signature of the deoxygenation by spectroscopic CPD measurements

a- Point spectroscopy measurements

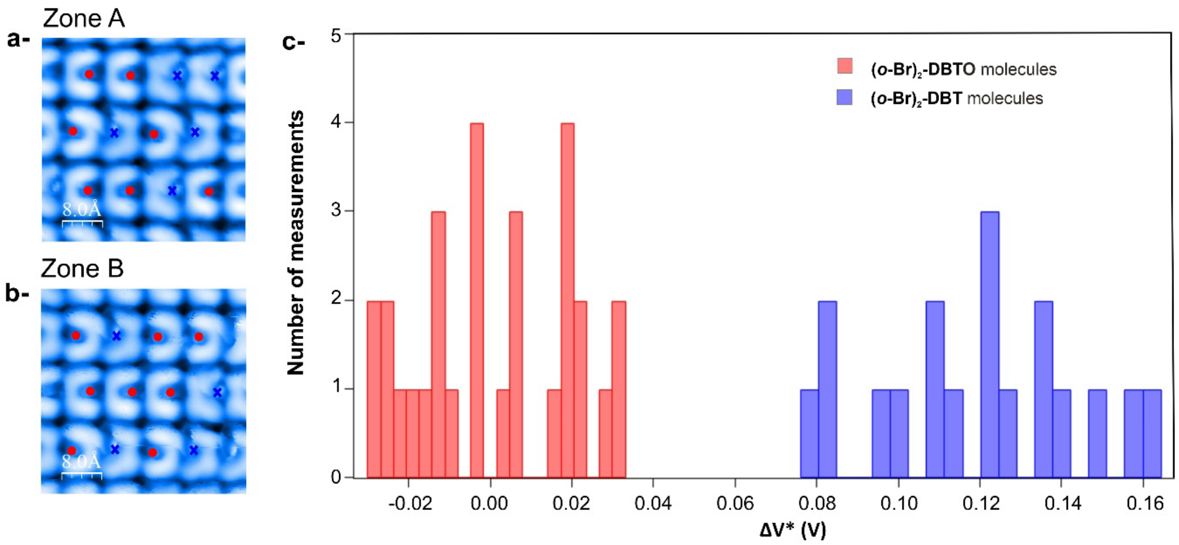

Fig. S9 | Bias spectroscopy study carried out on a total of 24 molecules (2 measurements per molecule). The molecules are divided into two groups: Zone A (a) and Zone B (b). Zone A contains 7 pristine (*o*-Br)$_2$-DBTO molecules (depicted by the red • symbol) and 5 deoxygenated (*o*-Br)$_2$-DBT ones (depicted by the blue *x* symbol), while Zone B presents 8 pristine (*o*-Br)$_2$-DBTO molecules and 4 deoxygenated (*o*-Br)$_2$-DBT ones. Bias spectroscopy measurements were performed above the sulfur atom of each molecule. For every molecule, two Δf(V) curves were acquired. In order to ensure that the molecules did not shift positions, STM pictures were captured both before and after each set of four curves. After collecting the data, a parabolic fit is obtained, and the local CPD (LCPD) is determined by the highest point of each curve. The LCPD values of pristine molecules are then averaged separately for each line and used as a reference (0). This step was necessary because maintaining consistent tip conditions for every line proved challenging and had small impact on the quantitative values of *Δf measured between the series of four curves. For this reason, not all lines have the same *Δf** values, but a higher LCPD for deoxygenated molecules than for pristine ones was consistently observed. Finally, the ΔV* is calculated for each bias spectroscopy measurement regarding the reference value. The results are displayed in the histogram of image (c).**

b- Spectroscopic grids

In the present example (Fig. S10), the grid consists of 60x30 pixels: the first molecule on the left is pristine **(*o*-Br)$_2$-DBTO**, while the other two are deoxygenated. Bias spectroscopy curves were recorded on each pixel of the preset grid, and the (*V**, *Δf**) values were then mapped in two dimensions over the corresponding area. Unlike the first procedure (point spectroscopy measurements, Fig. S9), a CO-tip is kept intact throughout the process. Atom tracking is utilized between two subsequent curves to guarantee that no x,y,z drift occurs. The atom tracking reference spot is chosen from a particular position on the grid (typically a maximum). The tip returns to its reference spot and corrects its x,y,z position from drift after every curve, such that the grid is recorded at *constant height* and with minimum lateral drift. Once the *Δf** map is obtained (Fig. S10a), the molecules are easily identifiable, as the carbon skeleton is clearly displayed. The *Δf** contrast is qualitatively similar to that obtained in dynamic STM mode (cf. Fig. S3b). Therefore, for each map obtained (**a**, **c**, **e,** and **g**) we can associate a model of the molecules shown in the corresponding images (**b**, **d**, **f,** and **h**).

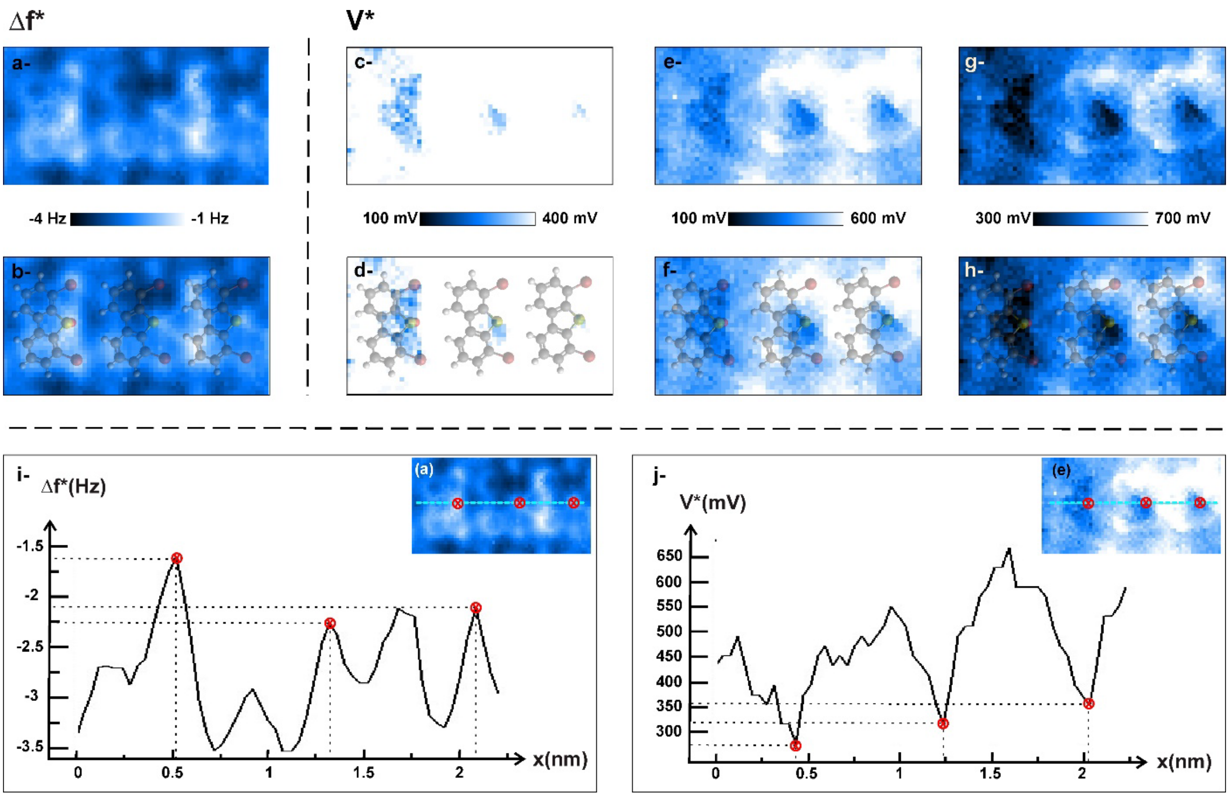

Fig. S10 | Bias spectroscopy study on a grid of 3x1 molecules (60x30 pixels). a-b, *Δf map. In (b), the molecular model has been overlaid to spot the locations of the molecules. c-h, corresponding *V** map reported with three increasing scales. In (d), (f), and (h), the molecular model has been overlaid to spot the locations of the molecules.**

The LCPD map (*V**) is presented on three different voltage scales (**c**,**d** : 100-400 mV - **e**,**f**: 100-600 mV - and **g**,**h**: 300-700 mV). The *V** value surrounding the sulfur atom of the first **(*o*-Br)$_2$-DBTO** molecule (pristine) is clearly lower than that of the sulfur atoms in the two **(*o*-Br)$_2$-DBT** molecules (deoxygenated), which is consistent with the curves shifting to higher *V** values

when the molecules are deoxygenated, as discussed in the point bias spectroscopy measurements (Fig. 4 of the main manuscript).

Figures (**i**) and (**j**) present cross-sections taken across the three molecules in the grid to illustrate variations in Δf^* and V^* values, respectively. In order to perform these cross-sections, figures (**a**) and (**e**) were taken as a reference: the blue line represents the area where the section was measured, and the red symbols represent the position of the sulfur atom of each molecule.

Fig. S10i demonstrates that the deoxygenated molecules exhibit lower Δf^* values than the pristine one. Two successive molecules (pristine and deoxygenated) show a difference of -0.65 Hz, (-0.57 Hz in average, when considering the 2nd deoxygenated molecule).

Fig. S10j displays the corresponding cross-section for V^* values, showing a +40 mV difference between pristine and deoxygenated molecules (+60 mV in average, when considering the 2nd deoxygenated molecule). These shifts in V^* values can be contrasted with those seen in Fig. S9 and Fig. 4. Although the differences are smaller due to different tip-surface distances, the trend remains consistent—deoxygenated molecules always exhibit higher V^* values than their pristine counterparts.

The LCPD map, that may be interpreted to first order as a charge state analysis of the molecules, reveals that the two deoxygenated molecules (in the center and on the right) have an overall larger LCPD compared to that of the pristine molecule (on the left). These molecules are therefore less/more positively/negatively charged compared to the pristine ones. So, the interactions between pristine and deoxygenated molecules can affect both their charge state and their local adsorption configuration, without however affecting the overall structure of the molecular domains.

Lastly, we notice that the carbon backbone of the second deoxygenated molecule seems to be lifted, similarly to what was observed in the dynamic STM measurement (cf. red dotted ellipses in Fig. S3 and caption). So, despite different kinds of imaging processes (<It> regulation vs. constant height), we image consistently the structural configuration of the adsorbed molecules.

To form a more statistically relevant set of data, several other grid spectroscopy experiments have been performed, which all show the same trend.

II. In-solution Investigations

1. Molecule synthesis

4,6-dibromodibenzo[*b*,*d*]thiophene 5-oxide; (*o*-Br)$_2$-DBTO:

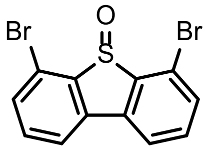

A solution of 4,6-dibromodibenzo[*b*,*d*]thiophene (1.03 g, 3.0 mmol, 1 equiv.) in dry DCM (20 mL) in a round bottom flask was cooled to -30 °C. $BF_3 \cdot OEt_2$ (3.0 mL, 24 mmol, 8 equiv.) was added dropwise and stirred for 10 minutes. A suspension of *m*CPBA (741 mg, 3.3 mmol, 1.1 equiv.) in DCM (5 mL) was then added dropwise. The cool bath was removed and the slurry reaction medium was stirred at room temperature for 24 hours. Aqueous NaOH (1M, 50 mL) and DCM (25 mL) were added, after vigorous stirring both phases were separated and the aqueous fraction was extracted once with DCM (25 mL). Combined organic phases were dried using Na_2SO_4, filtered (rinsing the solids with hot DCM thrice) and the volatiles were removed under vacuum. The crude reaction mixture was then dissolved in a minimum amount of boiling dichloromethane, colorless crystals (420 mg) were obtained upon cooling and standing at room temperature overnight. A second crop of crystals (210 mg) could be obtained from further recrystallization of the filtrate using boiling dichloromethane. Repeating this operation provided a third crop (60 mg). Overall yield: 690 mg, 1.93 mmol, 64%.

^{1}H NMR ($CDCl_3$, 300 MHz) δ_H7.71 (dd, *J* = 7.6, 0.9 Hz, 2H), 7.61 (dd, *J* = 8.0, 0.9 Hz, 2H), 7.46 (app. t, *J* = 7.8 Hz, 2H). **^{13}C{^{1}H} NMR (75 MHz, $CDCl_3$) δ_C** 144.4, 139.3, 134.3, 133.8, 123.3, 121.3. **HRMS (DCI-CH_4)** Calc'd for $C_{12}H_7OS$ $[M+H]^+$ 356.8584, found 356.8588. **FTIR** (neat) vmax/cm^{-1} 3055, 2993, 1575, 1557, 1462, 1421, 1105, 1044, 1032, 1025, 787. **Crystal data:** see section III. of the SI.

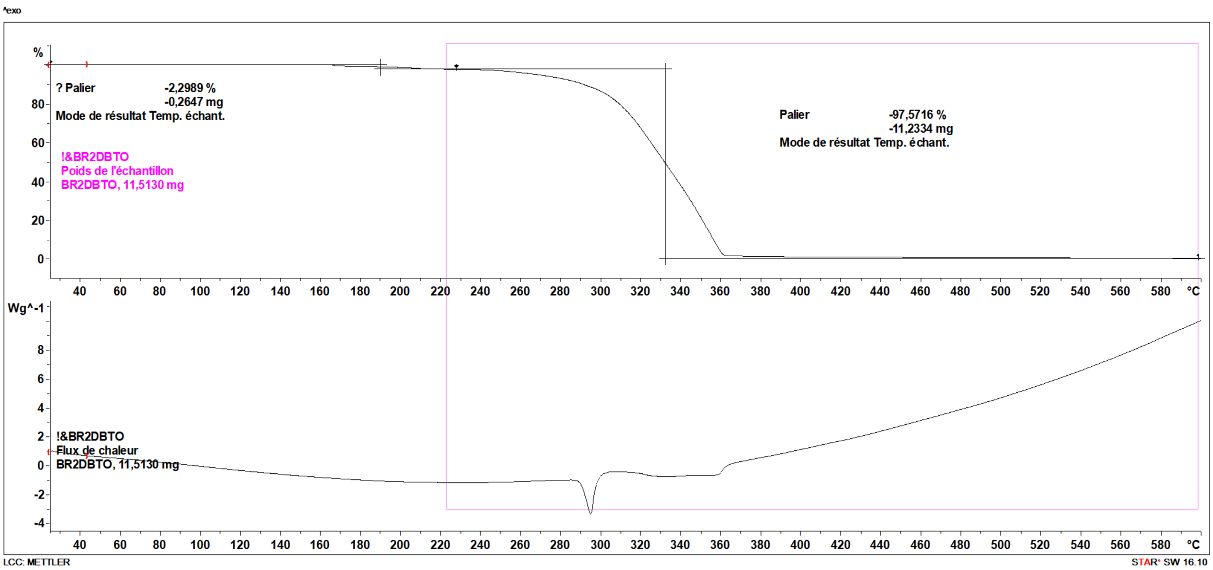

Fig. S11 | Thermogravimetric analysis of (*o*-Br)$_2$-DBTO (N_2 atmosphere, ramp from 25 to 600 °C, 10 °C/min)

2. Photoreactivity in solution

a- (*o*-Br)$_2$-DBTO photoreactivity in DCM solutions degassed by freeze-pump-thaw cycles:

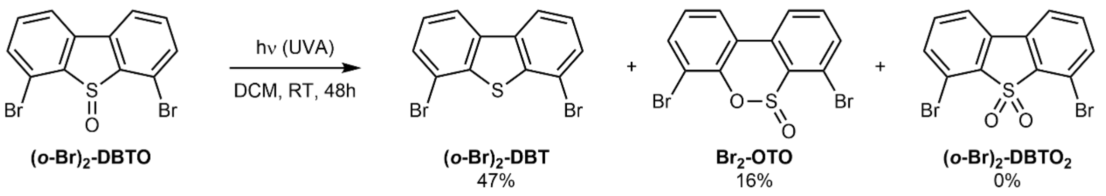

(*o*-Br)$_2$-DBTO (35.8 mg, 0.1 mmol) was dissolved in dry and freeze-pump-thaw degassed DCM (10 mL) under an inert atmosphere in a quartz Schlenk. The solution was then irradiated at room temperature using a UVA fluorescent tube (see Fig. S13 for the lamp emission spectrum) for 48 hours at which point TLC analysis showed complete conversion. The volatiles were removed in vacuo and the crude mixture was then purified over silica gel chromatography (gradient from 0 to 50% DCM: Pentane) yielding successively **(*o*-Br)$_2$-DBT** (16 mg, 0.047 mmol, 47%) and **Br$_2$-OTO** (6.1 mg, 0.016 mmol, 16%). The missing yield in sulfur derivatives may arise from photobleaching and tar deposition observed on the quartz glassware after extended irradiation.

b- (*o*-Br)$_2$-DBTO photoreactivity in DCM solutions degassed by argon sparging:

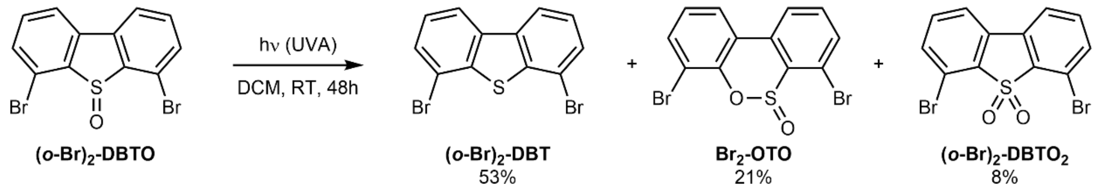

(*o*-Br)$_2$-DBTO (35.8 mg, 0.1 mmol) was dissolved in dry and argon-sparged DCM (10 mL) under an inert atmosphere in a quartz Schlenk. The solution was then irradiated at room temperature using a UVA fluorescent tube (see Fig. S13 for the lamp emission spectrum) for 48 hours at which point TLC analysis showed complete conversion. The volatiles were removed in vacuo and the crude mixture was then purified over silica gel chromatography (gradient from 0 to 50% DCM: Pentane) yielding successively **(*o*-Br)$_2$-DBT** (18 mg, 0.053 mmol, 53%), **Br$_2$-OTO** (8 mg, 0.021 mmol, 21%) and **(*o*-Br)$_2$-DBTO$_2$** (3 mg, 0.008 mmol, 8%).

c- Characterization of the photoproducts

4,6-dibromodibenzo[*b*,*d*]thiophene; (*o*-Br)$_2$-DBT
White amorphous solid. **Rf**: 0.9, 50/50 DCM/Pentane. **^{1}H NMR (CDCl$_3$, 300 MHz) δ_H** 8.07 (dd, *J* = 7.9, 0.9 Hz, 2H), 7.65 (dd, *J* = 7.7, 0.9 Hz, 2H), 7.38 (app. t, *J* = 7.8 Hz, 2H). *Data consistent with commercial sample.*

4,7-dibromodibenzo[*c*,*e*][1,2]oxathiine 6-oxide; Br$_2$-OTO
Colorless solid. **Rf**: 0.55, 50/50 DCM/Pentane. **^{1}H NMR (CDCl$_3$, 300 MHz) δ_H** 7.96 (dd, *J* = 7.9, 1.1 Hz, 1H), 7.90 (dd, *J* = 8.0, 1.4 Hz, 1H), 7.73 (dd, *J* = 8.0, 1.4 Hz, 1H), 7.68 (dd, *J* = 8.0, 1.0 Hz,

1H), 7.56 (app. t, *J* = 7.9 Hz, 1H), 7.23 (app. t, *J* = 7.9 Hz, 1H). **$^{13}C\{^1H\}$ NMR (151 MHz, $CDCl_3$) δ_C** 142.2, 137.7, 135.0, 133.7, 133.3, 129.6, 126.4, 124.2, 124.2, 122.0, 121.3, 116.2. **HRMS (DCI-CH_4)** Calc'd for $C_{12}H_7O_2S$ $[M+H]^+$ 372.8533, found 372.8531. **FTIR** (neat) vmax/cm^{-1} 3077, 2927, 1544, 1438, 1383, 1200, 1139, 1126, 1111, 1076, 1049, 1035, 845, 778, 717, 685. **Crystal data:** Crystals suitable for X-ray diffraction were obtained by diffusion of pentane in a chloroform solution of the titled compound; see section III. of the SI.

4,6-dibromodibenzo[*b*,*d*]thiophene 5,5-dioxide; (*o*-Br)$_2$-DBTO$_2$

Colorless solid. **Rf**: 0.25, 50/50 DCM/Pentane. **^1H NMR ($CDCl_3$, 600 MHz) δ_H** 7.72 (dd, *J* = 7.7, 0.8 Hz, 2H), 7.65 (dd, *J* = 8.0, 0.8 Hz, 2H), 7.49 (app. t, *J* = 7.8 Hz, 2H). **$^{13}C\{^1H\}$ NMR (151 MHz, $CDCl_3$) δ_C** 136.9, 135.0, 134.9, 113.1, 120.4, 117.7. **HRMS (DCI-CH_4)** Calc'd for $C_{12}H_7O_2S$ $[M+H]^+$ 372.8533, found 372.8531. **FTIR** (neat) vmax/cm^{-1} 3085, 3076, 1578, 1564, 1426, 1312, 1162, 780, 748, 664, 593. **Crystal data:** Crystals suitable for X-ray diffraction were obtained by slow cooling of a saturated hot solution of the titled compound in an ethanol/chloroform mixture; see section III. of the SI. *Data consistent with a sample prepared from 4,6-dibromodibenzo[b,d]thiophene mixed with 3 equivalents of mCPBA.*

d- Investigation of the photoreactivity of **Br$_2$-OTO** and **(*o*-Br)$_2$-DBTO$_2$**

Photoirradiation (UVA) of a dichloromethane solution of pure sultine **Br$_2$-OTO** did not lead to any conversion. Likewise, photoirradiation (UVA) of a dichloromethane solution of pure sulfone **(*o*-Br)$_2$-DBTO$_2$** did not lead to any conversion.

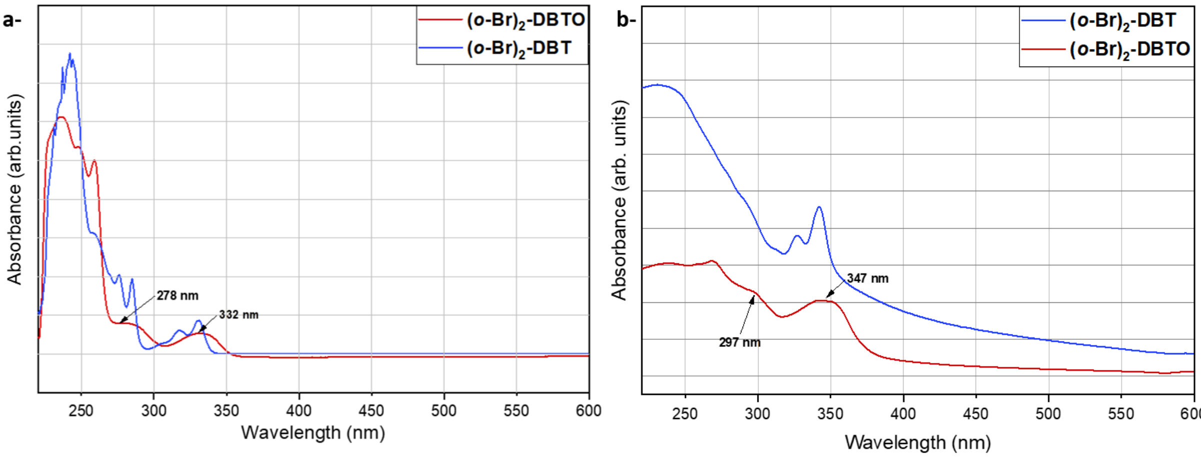

Fig. S12 | Absorption spectra of (*o*-Br)$_2$-DBTO and (*o*-Br)$_2$-DBT in solution (a) and in the solid state (b). a, The UV-vis optical absorption spectrum of (*o*-Br)$_2$-DBTO (red curve) in dichloromethane (DCM) solution (10^{-4} M) displays two wide absorption bands peaking at 332 and 278 nm, and a main feature in the 260-230 nm range. For the deoxygenated counterpart (*o*-Br)$_2$-DBT, the optical absorption spectrum (blue curve) in DCM (10^{-4} M) shows a main peak around 240 nm, with several peaks of lower intensity at λ_{max} = 276, 285, 318 and 332 nm. b, (*o*-Br)$_2$-DBTO and (*o*-Br)$_2$-DBT were dispersed in KBr at 2 %w/w, before being pressed at 6 tons yielding a glassy material directly used for UV-vis absorption experiments.

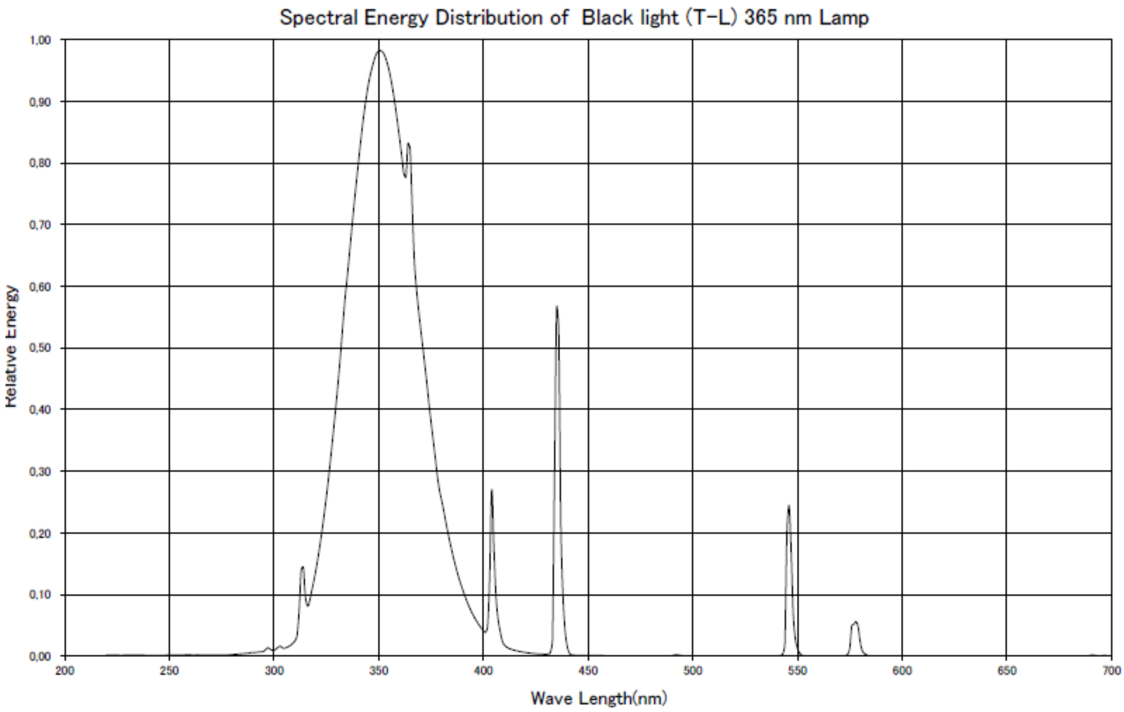

Fig. S13 | Emission spectrum of the UVA fluorescent tube used for in-solution photodeoxygenation reactions.

3. NMR spectra

a- 4,6-dibromodibenzo[*b*,*d*]thiophene 5-oxide; **(*o*-Br)$_2$-DBTO**:

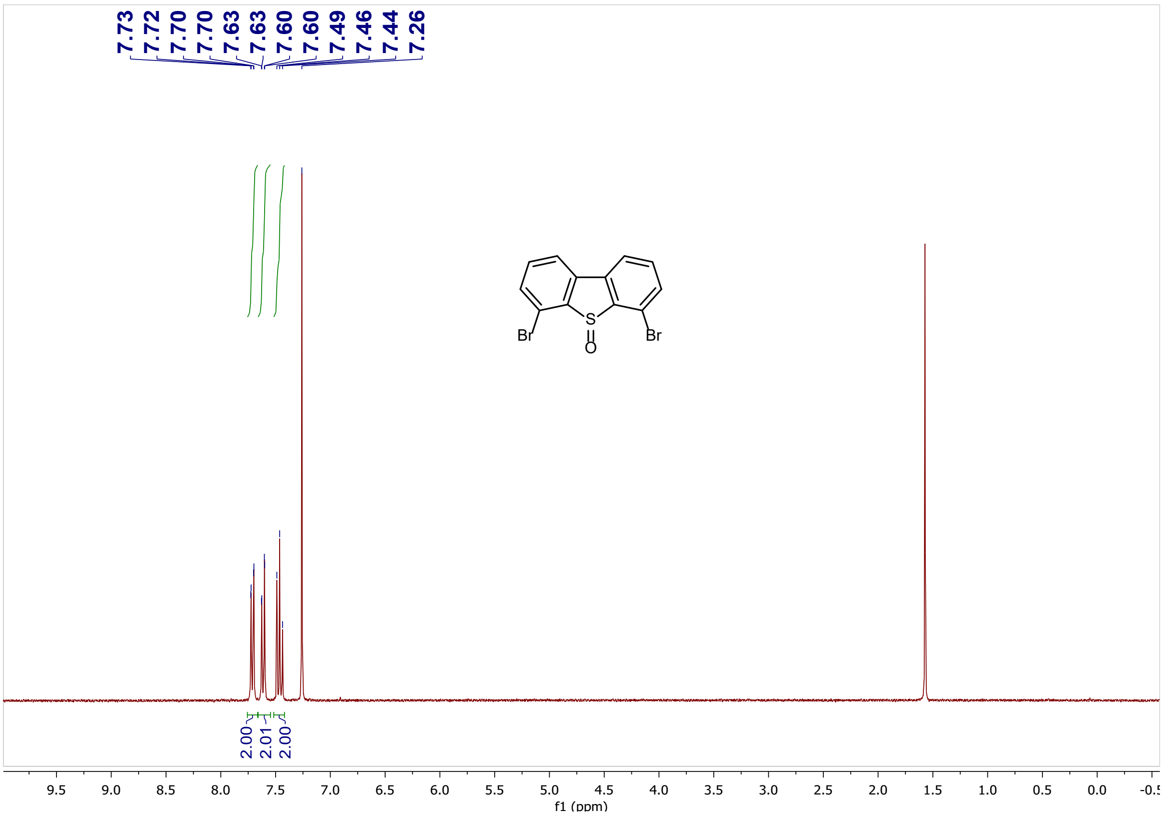

Fig. S14 | ^{1}H NMR spectrum of (*o*-Br)$_2$-DBTO (300 MHz, $CDCl_3$).

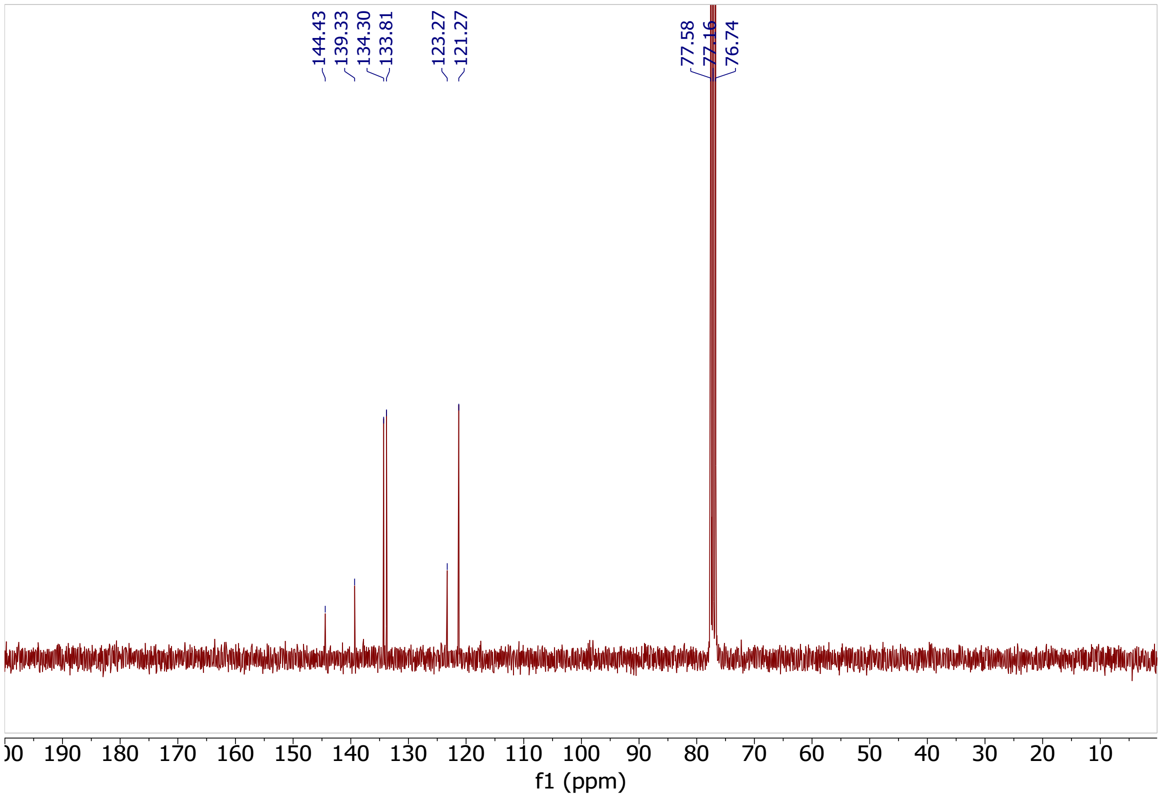

Fig. S15 | ^{13}C{^{1}H} NMR spectrum of (*o*-Br)$_2$-DBTO (75 MHz, $CDCl_3$).

b- 4,6-dibromodibenzo[*b*,*d*]thiophene; **(*o*-Br)$_2$-DBT**:

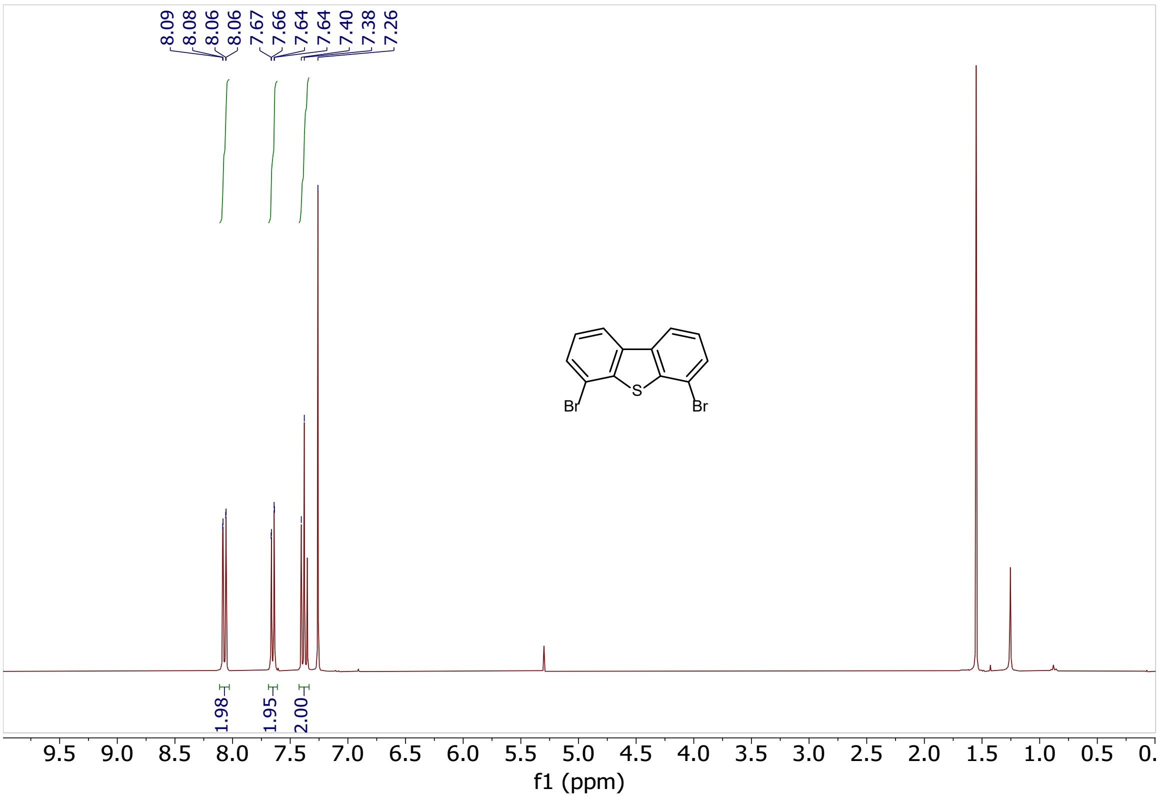

Fig. S16 | ^{1}H NMR spectrum of (*o*-Br)$_2$-DBT (300 MHz, $CDCl_3$).

c- 4,7-dibromodibenzo[*c*,*e*][1,2]oxathiine 6-oxide; **Br_2-OTO**:

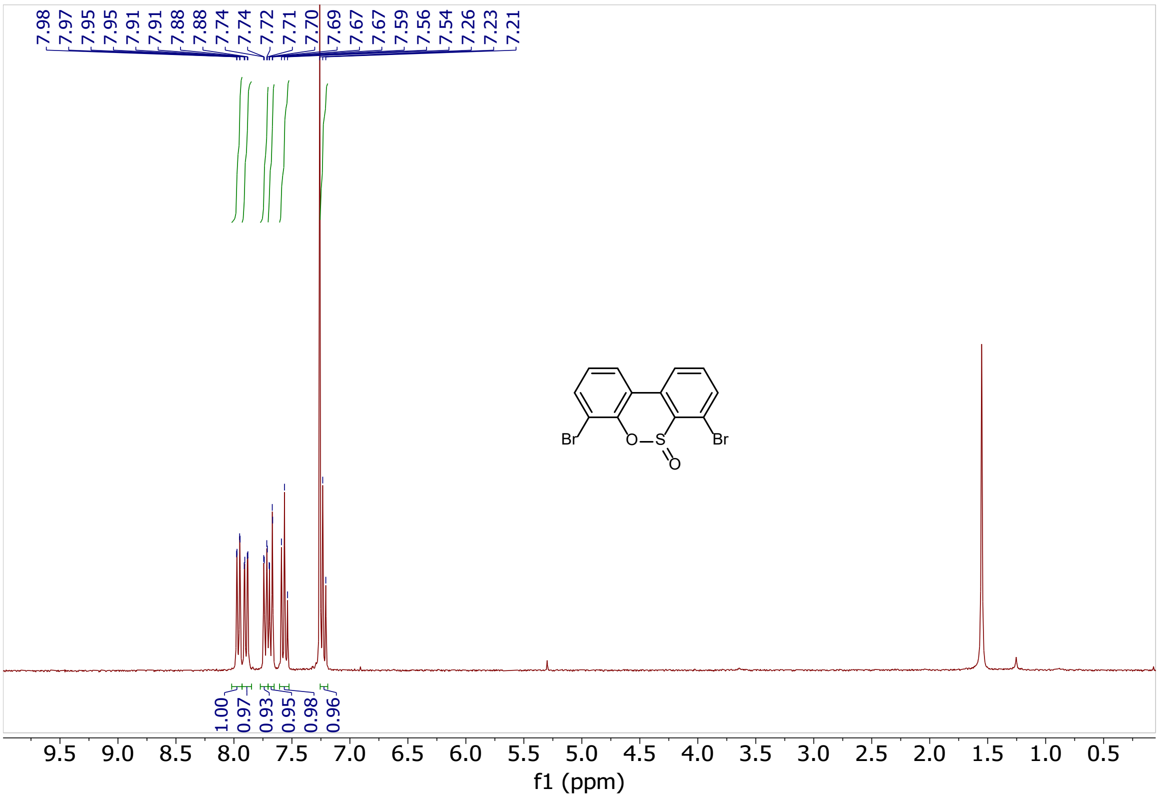

Fig. S17 | ^{1}H NMR spectrum of Br_2-OTO (300 MHz, $CDCl_3$).

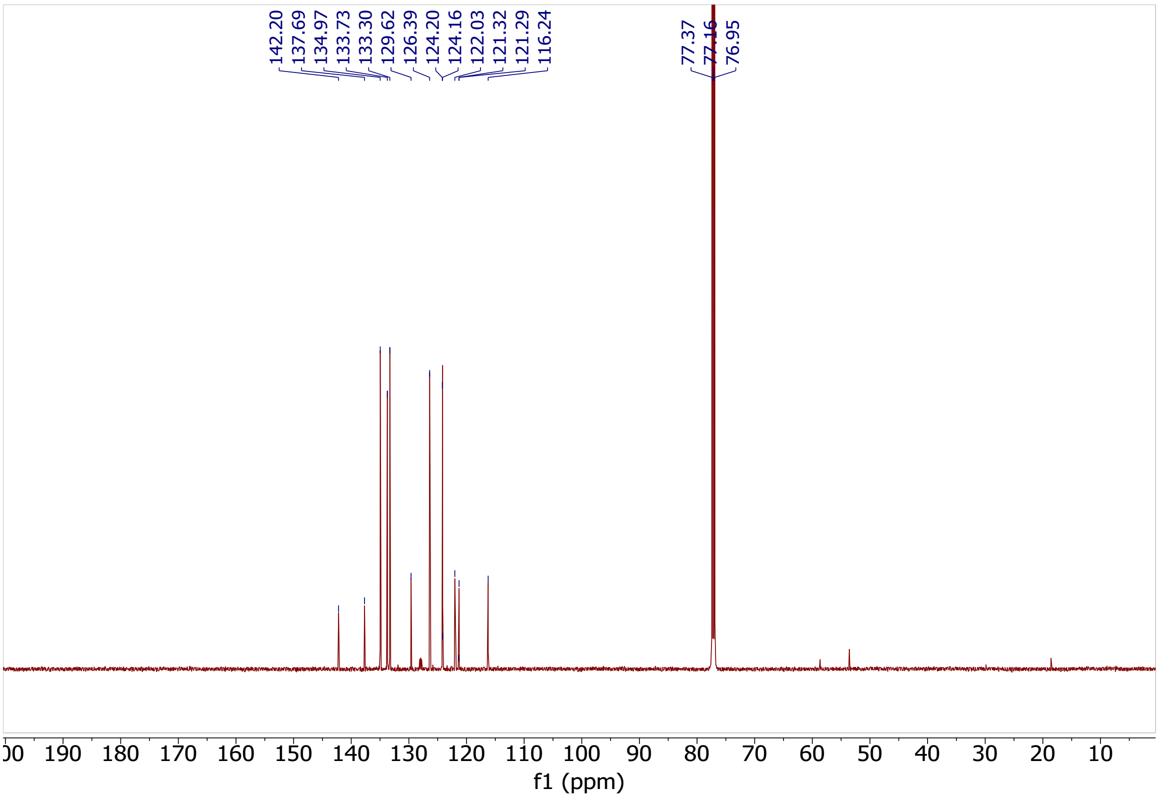

Fig. S18 | $^{13}C\{^1H\}$ NMR spectrum of Br_2-OTO (151 MHz, $CDCl_3$).

d- 4,6-dibromodibenzo[*b*,*d*]thiophene 5,5-dioxide; **(*o*-Br)$_2$-DBTO$_2$**:

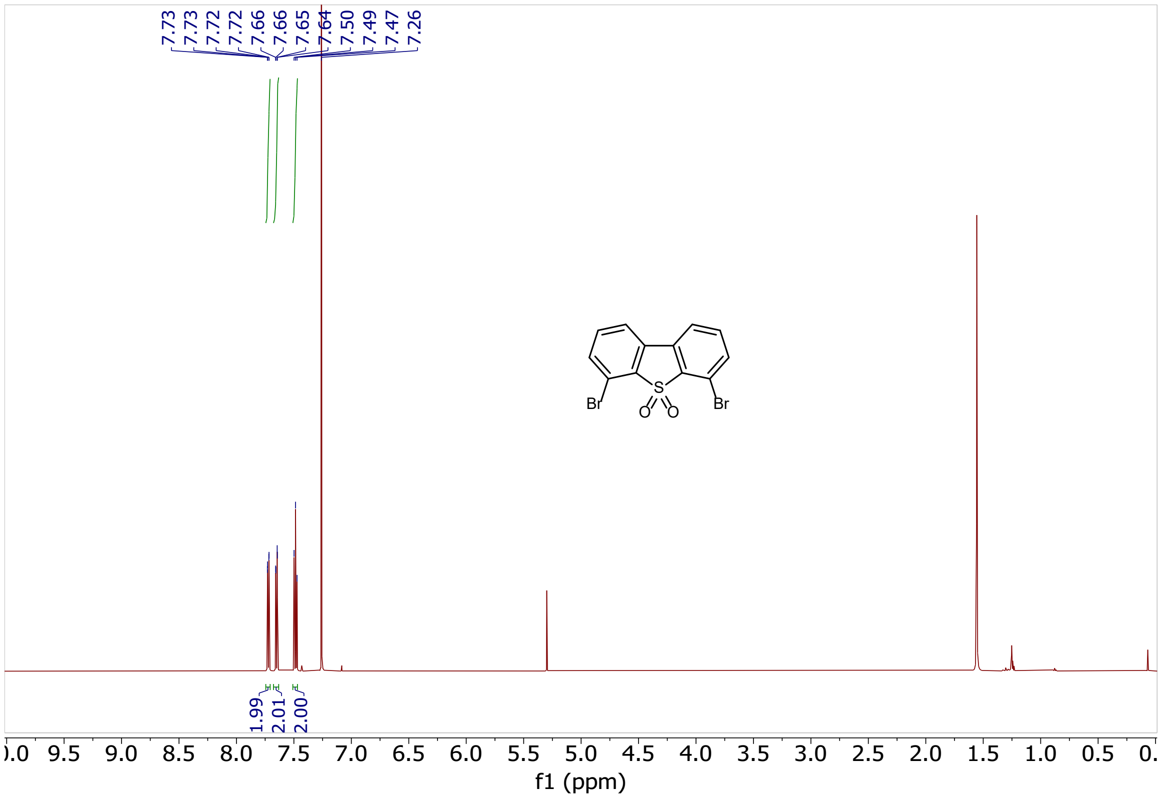

Fig. S19 | ^{1}H NMR spectrum of (*o*-Br)$_2$-DBTO$_2$ (600 MHz, CDCl$_3$).

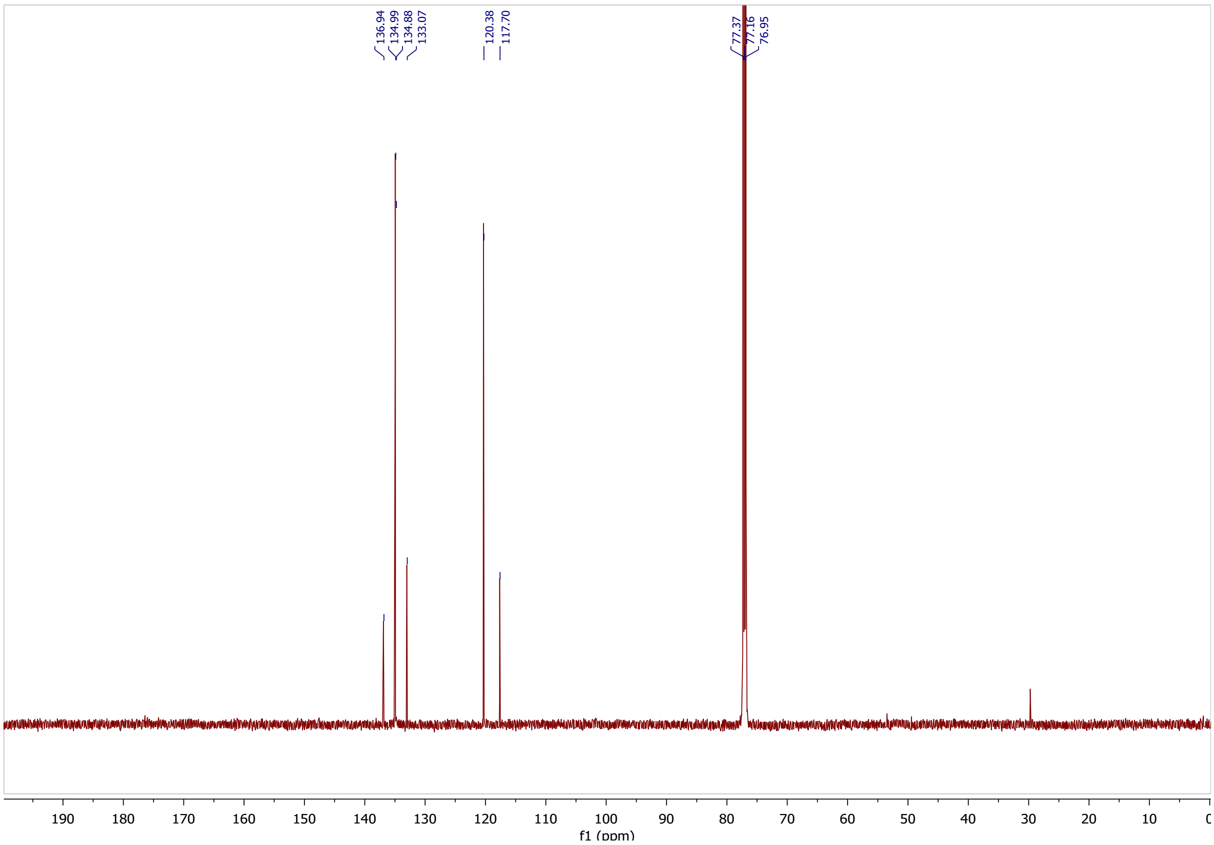

Fig. S20 | $^{13}C\{^1H\}$ NMR spectrum of (*o*-Br)$_2$-DBTO$_2$ (151 MHz, CDCl$_3$).

III. Crystallographic data

Single-crystal X-ray data were collected at low temperature (193(2)K) on a Bruker APEX II Quazar diffractometer equipped with a 30W air-cooled microfocus source (**(*o*-Br)$_2$-DBTO**) or on a Bruker D8 VENTURE diffractometer equipped with a PHOTON III detector (**Br$_2$-OTO** and **(*o*-Br)$_2$-DBTO$_2$**), using MoKα radiation (λ= 0.71037 Å). The structures were solved by intrinsic phasing method[5] and refined by full-matrix least-squares method on F^2. [6] All non-H atoms were refined with anisotropic displacement parameters and all the hydrogen atoms were refined isotropically at calculated positions using a riding model.

a- X-ray data for **(*o*-Br)$_2$-DBTO**

CCDC-2366290 contains the supplementary crystallographic data for this compound. These data can be obtained free of charge from The Cambridge Crystallographic Data Centre via https://www.ccdc.cam.ac.uk/structures.

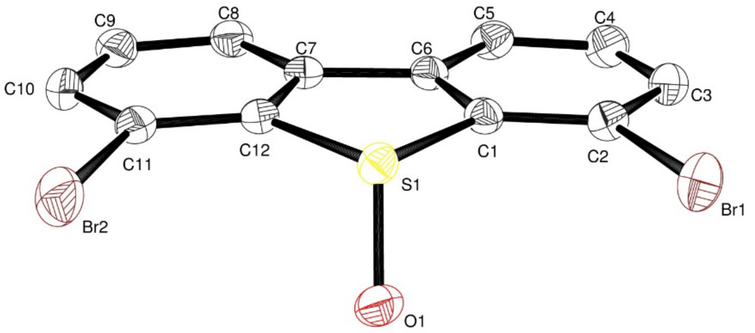

Fig. S21 | ORTEP view of the asymmetric unit of (*o*-Br)$_2$-DBTO, with thermal ellipsoids drawn at 50% probability (hydrogen atoms are omitted for clarity).

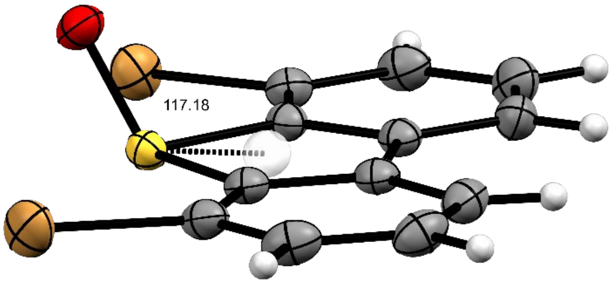

Fig. S22 | Pov-Ray image with ellipsoids at 50% probability showing the 117° angle between the SO bond and the aromatic core of (*o*-Br)$_2$-DBTO.

Table S1 | Crystal data and structure refinement for (*o*-Br)$_2$-DBTO.

Identification code	**(*o*-Br)$_2$-DBTO**
Empirical formula	C12 H6 Br2 O S
Formula weight	358.05
Temperature	193(2) K
Wavelength	0.71073 A
Crystal system, space group	Monoclinic, P 21/n
Unit cell dimensions	a = 7.6547(4) A alpha = 90 deg.
	b = 8.0798(4) A beta = 95.204(3) deg.
	c = 18.3175(11) A gamma = 90 deg.
Volume	1128.24(11) A^3
Z, Calculated density	4, 2.108 Mg/m^3
Absorption coefficient	7.341 mm^-1
F(000)	688
Crystal size	0.200 x 0.160 x 0.100 mm
Theta range for data collection	2.801 to 29.627 deg.
Limiting indices	-10<=h<=10, -11<=k<=10, -25<=l<=24
Reflections collected / unique	15090 / 3176 [R(int) = 0.0292]
Completeness to theta = 25.242	99.9 %
Refinement method	Full-matrix least-squares on F^2
Data / restraints / parameters	3176 / 0 / 145
Goodness-of-fit on F^2	1.026
Final R indices [I>2sigma(I)]	R1 = 0.0222, wR2 = 0.0492
R indices (all data)	R1 = 0.0294, wR2 = 0.0512
Largest diff. peak and hole	0.379 and -0.495 e.A^-3

b- X-ray data for **Br$_2$-OTO**

CCDC-2366291 contains the supplementary crystallographic data for this compound. These data can be obtained free of charge from The Cambridge Crystallographic Data Centre via https://www.ccdc.cam.ac.uk/structures.

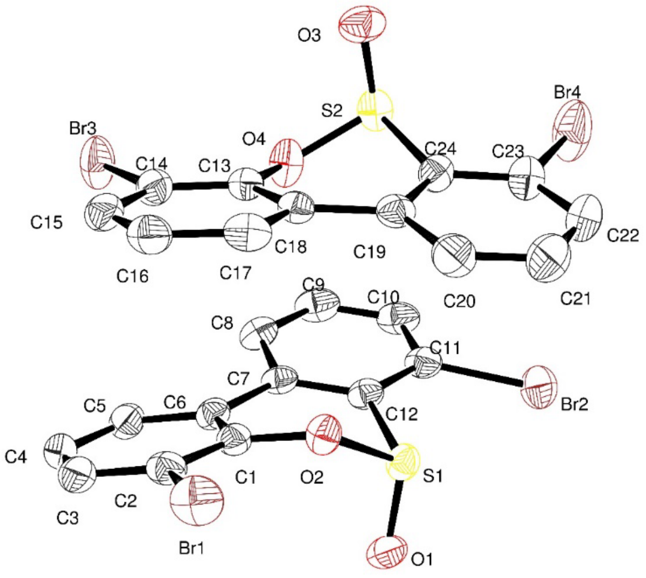

Fig. S23 | ORTEP view of the asymmetric unit of Br$_2$-OTO, with thermal ellipsoids drawn at 50% probability (hydrogen atoms are omitted for clarity).

Table S2 | Crystal data and structure refinement for Br_2-OTO

Identification code	**Br_2-OTO**
Empirical formula	C12 H6 Br2 O2 S
Formula weight	374.05
Temperature	193(2) K
Wavelength	0.71073 A
Crystal system, space group	Monoclinic, P 21/c
Unit cell dimensions	a = 14.908(6) A alpha = 90 deg.
	b = 14.778(9) A beta = 109.731(10) deg.
	c = 11.443(4) A gamma = 90 deg.
Volume	2373.1(18) A^3
Z, Calculated density	8, 2.094 Mg/m^3
Absorption coefficient	6.991 mm^-1
F(000)	1440
Crystal size	0.160 x 0.080 x 0.060 mm
Theta range for data collection	2.757 to 25.938 deg.
Limiting indices	-18<=h<=18, -18<=k<=18, -14<=l<=12
Reflections collected / unique	41559 / 4603 [R(int) = 0.0582]
Completeness to theta = 25.242	99.9 %
Refinement method	Full-matrix least-squares on F^2
Data / restraints / parameters	4603 / 0 / 307
Goodness-of-fit on F^2	1.033
Final R indices [I>2sigma(I)]	R1 = 0.0325, wR2 = 0.0754
R indices (all data)	R1 = 0.0453, wR2 = 0.0817
Largest diff. peak and hole	1.388 and -1.064 e.A^-3

c- <u>X-ray data for **(*o*-Br)$_2$-DBTO$_2$**</u>

CCDC-2366292 contains the supplementary crystallographic data for this compound. These data can be obtained free of charge from The Cambridge Crystallographic Data Centre via https://www.ccdc.cam.ac.uk/structures.

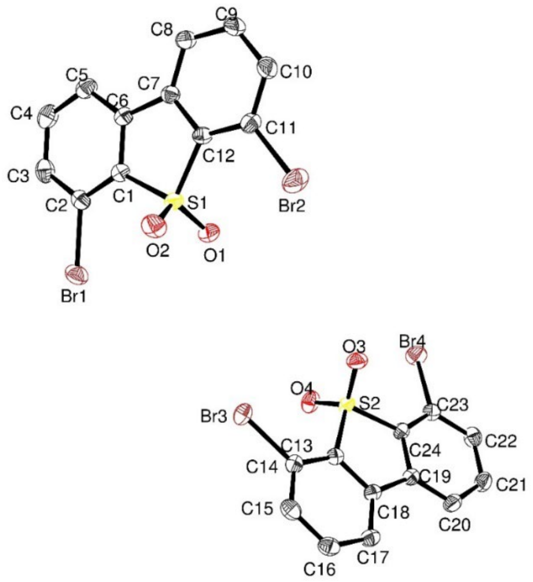

Fig. S24 | ORTEP view of the asymmetric unit of (*o*-Br)$_2$-DBTO$_2$, with thermal ellipsoids drawn at 50% probability (hydrogen atoms are omitted for clarity).

Table S3 | Crystal data and structure refinement for (*o*-Br)$_2$-DBTO$_2$.

Identification code	**(o-Br)$_2$-DBTO$_2$**
Empirical formula	C12 H6 Br2 O2 S
Formula weight	374.05
Temperature	193(2) K
Wavelength	0.71073 A
Crystal system, space group	Triclinic, P -1
Unit cell dimensions	a = 7.7340(9) A alpha = 116.240(4) deg.
	b = 12.7252(16) A beta = 99.561(5) deg.
	c = 13.8282(18) A gamma = 97.142(5) deg.
Volume	1173.2(3) A^3
Z, Calculated density	4, 2.118 Mg/m^3
Absorption coefficient	7.071 mm^-1
F(000)	720
Crystal size	0.180 x 0.080 x 0.020 mm
Theta range for data collection	2.740 to 33.224 deg.
Limiting indices	-11<=h<=11, -19<=k<=19, -21<=l<=21
Reflections collected / unique	47884 / 8940 [R(int) = 0.0525]
Completeness to theta = 25.242	99.9 %
Refinement method	Full-matrix least-squares on F^2
Data / restraints / parameters	8940 / 0 / 307
Goodness-of-fit on F^2	1.002
Final R indices [I>2sigma(I)]	R1 = 0.0308, wR2 = 0.0632
R indices (all data)	R1 = 0.0547, wR2 = 0.0710
Largest diff. peak and hole	0.454 and -0.846 e.A^-3

References

1. Hapala, P., Temirov, R., Tautz, F. S. & Jelínek, P. Origin of high-resolution IETS-STM images of organic molecules with functionalized tips. *Phys. Rev. Lett.* **113**, 226101 (2014).

2. Hapala, P. *et al.* Mechanism of high-resolution STM/AFM imaging with functionalized tips. *Phys. Rev. B* **90**, 085421 (2014).

3. Hanwell, M. D. *et al. Open Access Avogadro: An Advanced Semantic Chemical Editor, Visualization, and Analysis Platform*. *J. Cheminf.* vol. 4 http://www.jcheminf.com/content/4/1/17 (2012).

4. Sun, X., Felicissimo, M. P., Rudolf, P. & Silly, F. NaCl multi-layer islands grown on Au(111)-(22 × √3) probed by scanning tunneling microscopy. *Nanotechnology* **19**, 495307 (2008).

5. ShelXT, G. M. Sheldrick, University of Göttingen, *Acta Crystallogr.* Sect. A, **71**, 3-8 (2015).

6. ShelXL, G. M. Sheldrick, University of Göttingen, *Acta Crystallogr.* Sect. C **71**, 3-8 (2015).
